# Antimicrobial Coatings for Medical Textiles via Reactive Organo-Selenium Compounds

**DOI:** 10.3390/molecules28176381

**Published:** 2023-08-31

**Authors:** Ejajul Hoque, Phat Tran, Unique Jacobo, Nicholas Bergfeld, Sanjit Acharya, Julia L. Shamshina, Ted W. Reid, Noureddine Abidi

**Affiliations:** 1 Fiber and Biopolymer Research Institute, Department of Plant and Soil Science, Texas Tech University, Lubbock, TX 79409, USA; 2 Department of Microbiology and Immunology, Texas Tech University Health Sciences Center, Lubbock, TX 79430, USA; 3 Honors College, Texas Tech University, Lubbock, TX 79409, USA; 4 Ophthalmology and Visual Sciences, Texas Tech University Health Sciences Center, Lubbock, TX 79430, USA

**Keywords:** reactive organo-selenium compounds, medical textiles, bactericidal activity, fungicidal activity, cationized cotton

## Abstract

Bleached and cationized cotton fabrics were chemically modified with reactive organoselenium compounds through the nucleophilic aromatic substitution (S_N_Ar) reaction, which allowed for organo-selenium attachment onto the surface of cotton fabrics via covalent bonds and, in the case of the cationized cotton fabric, additional ionic interactions. The resulting textiles exhibited potent bactericidal activity against *S. aureus* (99.99% reduction), although only moderate activity was observed against *E. coli*. Fabrics treated with reactive organo-selenium compounds also exhibited fungicidal activities against *C. albicans*, and much higher antifungal activity was observed when organo-selenium compounds were applied to the cationized cotton in comparison to the bleached cotton. The treatment was found to be durable against rigorous washing conditions (non-ionic detergent/100 °C). This paper is the first report on a novel approach integrating the reaction of cotton fabrics with an organo-selenium antimicrobial agent. This approach is attractive because it provides a method for imparting antimicrobial properties to cotton fabrics which does not disrupt the traditional production processes of a textile mill.

## 1. Introduction

One of the markets for technical textiles that is growing the fastest is the market for *medical* textiles. Medical textiles are used in medical settings to treat wounds or medical conditions, or to provide an appropriate atmosphere for clinical therapy. Medical textiles range from non-woven fabric components of healthcare and hygiene items (diapers and feminine hygiene products) to non-implantable materials (wound dressings, bandages, gauzes, and surgical disposables) and highly specialized textiles used inside the human body. These include extracorporeal devices (such as prostheses) and implantable materials (high-value products utilized for blood filtration, sutures, tissue growth scaffolds, and vascular grafts) [1]. The global market for medical textiles is enormous: its size was estimated to be USD 14.42 billion in 2020 and is projected to reach USD 19.66 billion in 2026, at a compound annual growth rate (CAGR) of 5.3% [2].

Among medical textiles, surgical and hospital textiles used by medical workers are intended to reduce the spread of infection between patients and healthcare personnel and protect them from the transmission of bacteria that can persist on clinical clothing for several days and even after washing [3]. These products benefit from the use of *antimicrobial* medical textiles that, upon coming into direct contact with endogenous bacteria, have a potential to inhibit the bacterial growth on their surfaces and reduce cross-infections in primary care settings [4]. Because an open wound provides an ideal environment for bacterial and fungal proliferation that might trigger wound inflammation, antimicrobial wound dressings are another type of antimicrobial textile product that are effective in reducing the risks of infection [5,6]. Antimicrobial fabrics are also used as hospital bed sheets, curtains, and patient clothing, etc., to reduce cross-infection [7]. Outside of hospital settings, antimicrobial textiles are being used in regular clothes [8] and sportswear [9]. 

Antibacterial textiles are traditionally produced by the treatment of textiles with a solution and/or suspension of an antimicrobial agent, which is applied onto fabric via simple dip-coating and/or spraying techniques. The fabric itself is made from non-toxic, non-allergic, non-carcinogenic, and sterilizable fibers, for which the properties important for the final product manufacture—strength, flexibility, elongation, absorbency, and biodegradability—are already known. For a long time, cotton and its derivatives have been used extensively for this purpose. However, while cotton is recognized for its diverse use, durability, and comfort [10], its hydrophilic nature could create an ideal habitat for the development of bacteria. Being made entirely of cellulose [11], daily textiles produce a warm, humid milieu on the skin, encouraging bacterial proliferation. Sweat discharges and skin peeling, etc., contribute to bacterial growth [12], and the bacteria can cause a foul smell, discoloration, fabric degradation, and skin rashes and infection.

Common textile antimicrobial agents include quaternary ammonium compounds (QACs), triclosan, chitosan, polyhexamethylene biguanide (PHMB), *N*-halamines, and metal nanoparticles (NPs). Despite having numerous advantageous qualities, QACs easily leach from fabric. In terms of their chemical structure, QACs lack reactive functional groups that would allow for covalent attachment to the fibers [13]. Chitosan demonstrates its antibacterial activity only in acidic media because its antibacterial effect is caused by a quaternary ammonium (–NH_3_^+^) group, which gets protonated at pH < 6.5 [14]. Chitosan also binds poorly to cellulose fibers. PHMB is acutely toxic for human keratocytes [15]. A significant quantity of adsorbed Cl may accumulate on the surface of fibers after the *N*-halamine treatment of textiles, which may cause unpleasant odors or even the discoloration of clothes [16]. Metal NPs are toxic and are able to penetrate the skin, lungs, and digestive system, contributing to the production of free radicals, which can induce cell damage [17]. In addition, bacterial resistance is a common drawback for all the aforementioned antibacterial agents. 

Recently, a selenium (Se)-based family has been explored for its anticancer [18], antioxidant [19], and antibacterial [20,21,22] activities. In respect to its antibacterial activity, which is of interest to this particular work, bacteria possess thiol-dependent enzyme systems as a part of their Reactive Oxygen Species (ROS) defense. Low-molecular-weight thiols, such as thioredoxin (Trx) and glutathione, etc., act as antioxidants in bacterial cells, helping them to survive oxidative damage. The antibacterial mechanism of organo-selenium compounds is based on their thiol-oxidizing ability and inhibition of the thiol-dependent ROS defense. Namely, the organoselenium anion (R–Se^−^) continuously donates an electron to the molecular oxygen in the bacteria cell, with the formation of a superoxide anion radical (O_2_^●−^) and organoselenium radical (R–Se^●^), which, in turn, react with the low-molecular-weight (MW) thiolates (e.g., the anion of glutathione (GS^−^)) [23], with the formation of Se–S bonds, excluding thiols from the ROS defense [24]. 

It is noteworthy that, while humans also possess a thiol-dependent enzyme system, the reactivity of organoselenium compounds depends on the MW of the thiol, which is significantly larger for mammals [24,25,26]. For instance, the MW of mammalian thioredoxin (Trx) is 55 kDa, whereas the MW of bacterial Trx is 35 kDa [27]. In addition, bacterial Trx is Se-free [27], while mammalian Trx is a Se-containing enzyme able to react with OS compounds to form Se–Se bonds and actually enhance the ROS defense [24]. 

**Figure 1:**
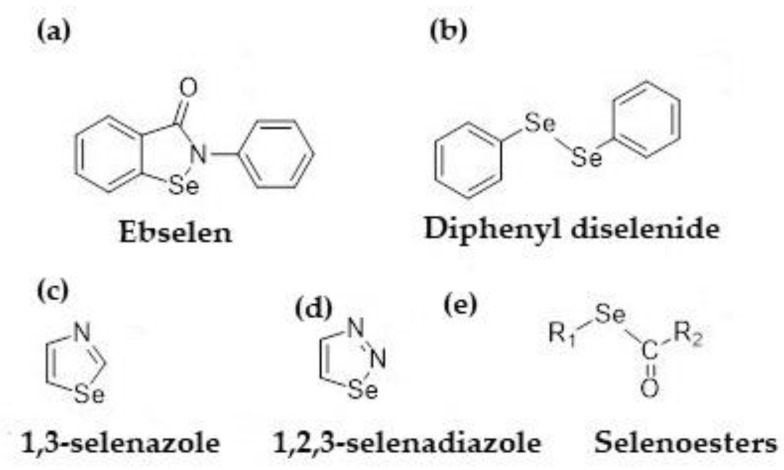
Organo-selenium compounds with antibacterial activity. (**a**) Ebselen, (**b**) Diphenily diselenide, (**c**) 1,3-selenazole, (**d**) 1,2,3-selenadiazole, (**e**) Selenoesters.

Several generations of organoselenum compounds (OS) have been synthesized and tested against various microbes for decades [28,29]. The first OS compound was diethyl selenide [30]. Later developed examples include Ebselen and its derivatives (Figure 1a), active against *S. aureus*, *Helicobacter pylori*, and *Mycobacterium tuberculosis* [31,32,33,34]. Diphenyl diselenide (Figure 1b) is known to be effective against *Aspergillus* sp. [35], *Fusarium* sp. [36], *Cryptococcus* sp. [37], *Candida glabrata* [38], *Trichosporon asahii* [39], *S. aureus*, and *S. epidermidis* [40]. Selenazoles (Figure 1c) and their derivatives have been shown to exhibit antibacterial activity against the fungi *Candida albicans* and *Cryptococcus neoformans* [41,42,43], and have been used for the functionalization of known antibiotics to enhance their activity [44]. Selenediazole and its derivatives (Figure 1d) have exhibited antimicrobial action against *E. coli*, *S. aureus*, and *Mycobacterium tuberculosis* [45]. Another class of selenium compounds, selenoesters (Figure 1e) such as methylketone selenoester, have demonstrated promising antibacterial activity against *S. aureus*, *Enterococcus faecalis*, *Chlamydia trachomatis* [46], *Salmonella enterica serovar Typhimurium*, and *Candida* sp. [47,48]. These organoselenum compounds have been used as coatings for polyester bandages [49], scleral buckles [50], contact lenses [51], catheter tubing [52], and even cotton socks [53]. 

**Figure 2:**
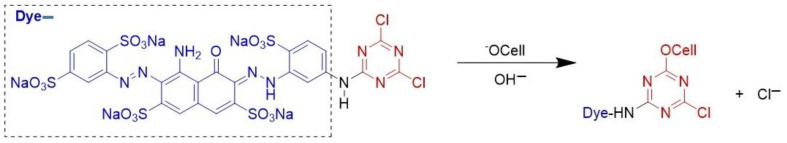
Reaction of C.I. Reactive Blue 109 with cellulose.

To the best of our knowledge, none of these studies have investigated the application of reactive OS compounds, i.e., OS compounds possessing a reactive functional group for covalent attachment to cellulose, the main component of cotton fabric. At the same time, reactive dyes, a common class of textile dyes, are often applied to cotton and react with the hydroxyl groups of cellulose, forming covalent bonds in the presence of alkali via a nucleophilic substitution reaction [54]. These dyes form a permanent attachment to the fiber with a high degree of wash fastness. An example of such a reactive dye is C.I. Reactive Blue 109 (sodium (Z)-5-amino-3-(2-(5-((4,6-dichloro-1,3,5-triazin-2-yl)amino)-2-sulfonatophenyl)hydrazineylidene)-6-((E)-(2,5-disulfonatophenyl)diazenyl)-4-oxo-3,4-dihydronaphthalene-2,7-disulfonate), which contains a 4,6-dichloro-1,3,5-triazin-2-yl fragment. The 4,6-dichloro-1,3,5-triazin-2-yl fragment reacts with cellulose via the nucleophilic aromatic substitution (S_N_Ar) reaction, resulting in the substitution of chlorine atoms with cellulose and the formation of a covalent bond between the cellulose and the dye (Figure 2).

We hypothesized that the incorporation of a 4,6-dichloro-1,3,5-triazin-2-yl fragment into the structure of OS compounds would allow us to reactively attach the OS compounds onto the cotton fabric, and that conditions suitable for dyeing would be applicable for creating a covalent bond between the OS compounds and the cotton cellulose. In addition, chloro- and dichloro-1,3,5-triazin-2-yl-containing molecules are known to exhibit anti-bacterial [55,56] and anti-fungal [57] activities that might be synergistic to selenium activity.

The uptake of reactive dyes can be increased by the means of the cationization of cotton (CC) to introduce cationic sites able to attract negatively charged and/or aromatic dye molecules [54], in addition to covalent attachment. Therefore, we decided to also utilize CC textiles and compare the results with bleached cotton (BC) fabric used as a control.

**Figure 3:**
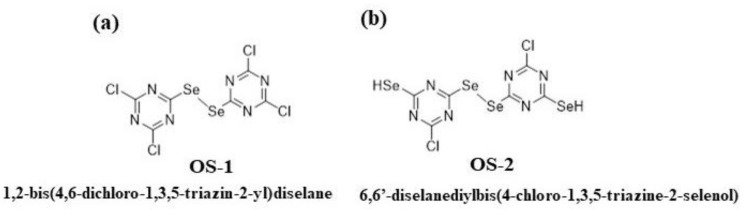
OS compounds, 1,2-bis(4,6-dichloro-1,3,5-triazin-2-yl)diselane (OS-1, (**a**)) and 6,6′-diselanediylbis(4-chloro-1,3,5-triazine-2-selenol) (OS-2, (**b**)).

Attach Chem Inc. (Lubbock TX) synthesized two reactive OS compounds (patent protected, OS-1 and OS-2, Figure 3) for textile application, which differed in their number of Se- atoms and number of potential reactive sites. The objective of this research was to investigate whether the 4-chloro-1,3,5-triazin-2-yl and 4,6-dichloro-1,3,5-triazin-2-yl fragments of the OS compounds were able to react with the cotton cellulose under an alkaline pH, and if the resulting fabric would exhibit antimicrobial properties against Gram-positive (*S. aureus*), Gram-negative (*E. coli*) bacteria, and yeast (*Candida albicans*).

## 2. Results

### 2.1. Fabric Cationization

Among the various compounds that have been utilized to produce cationic cotton, a commercially available CR-2000, with the chemical name (3-chloro-2-hydroxypropyl)trimethylammonium chloride (CHPTAC), is a well-established and widely studied cationizing agent [58]. Upon its covalent attachment to cellulose, CHPTAC introduces positive quaternary ammonium groups onto the cotton fabric surface. Our group has previously utilized CHPTAC for the cationization of cotton fabrics [59]; the same protocol for cationization was used in the current study. 

**Figure 4:**
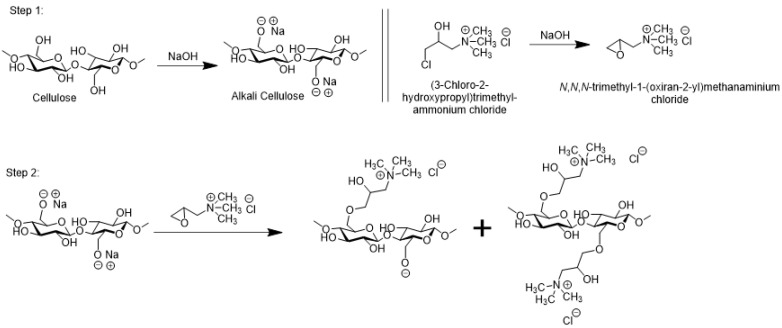
Mechanism of the reaction of CHPTAC with cotton cellulose under alkaline conditions.

The possible two-step mechanism of the reaction of CHPTAC with BC under alkaline conditions is outlined in Figure 4. In the first step, the cellulose of the BC textile is transformed into alkali cellulose and the CHPTAC is converted into its reactive epoxide form. In the second step, the alkali cellulose reacts with the epoxide to produce cationized cotton (CC) fabric. The unreacted epoxide is hydrolyzed with water, with the formation of 2,3-dihydroxy-*N*,*N*,*N*-trimethylpropanaminium chloride that is washed out from the fabric after the reaction.

### 2.2. Preparation of Organoselenium Textiles

#### 2.2.1. Preparation

**Figure 5:**
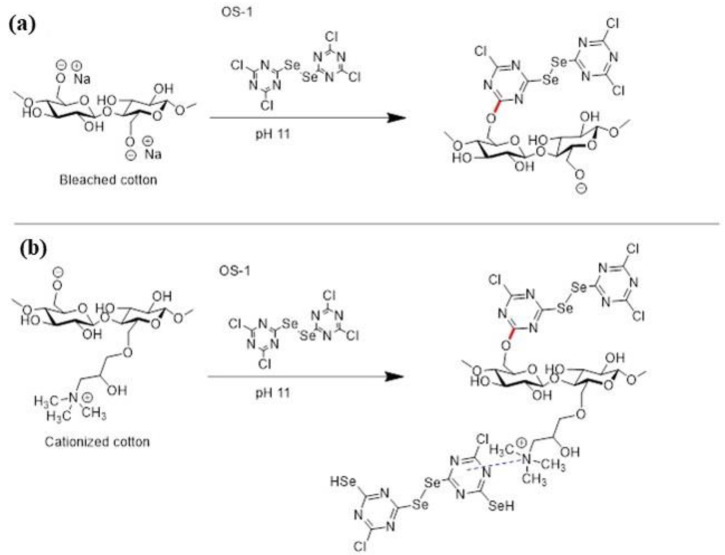
Representative reaction between the cotton fiber and the OS-1 compound: (**a**) bleached cotton fiber and (**b**) cationized cotton fiber. The red color represents the newly formed covalent bond while the blue color represents ionic bond.

After the fabric cationization, the OS compounds were applied to BC and CC fabrics (see Experimental). For the application of the OS compounds to the BC fabric, the fabrics were treated with aqueous solutions of the OS compounds at different concentrations (1%, 3%, and 5%), in the presence of Na_2_CO_3_ and Glauber’s salt (Na_2_SO_4_). Under alkaline conditions, cellulose-based nucleophile Cell-O^−^ entered into a S_N_Ar reaction with the reactive 4-chloro-1,3,5-triazin-2-yl chloride fragments of the OS compounds. Basic Na_2_CO_3_ was added to the treatment bath for the adjustment of the pH, and, in order to enhance the fixation of the OS compounds to the BC fabric, Glauber’s salt was used to neutralize the negative zeta potential of the cotton fabric and facilitate the uptake of the OS compounds. Figure 5a illustrates the reaction between the BC fiber and OS-1 compound; the reaction with OS-1 proceeded through the same mechanism and is not shown here. 

The protocol was similar for the application of the OS compounds to the CC fabric, with the exception that no Na_2_SO_4_ was required due to the presence of positive cationic sites. Figure 5b illustrates the reaction between the CC fiber and OS-2 compound. In addition to the formation of a covalent bond between the OS compounds and the cellulose of the CC fabric as a result of the S_N_Ar reaction, ionic interactions between the quaternary ammonium group of the CC and the aromatic ring of the OS compounds were also established [60], allowing for a higher fixation of the OS compounds on the CC than that on the BC, as is evident in the following sections.

#### 2.2.2. Appearance

**Figure 6:**
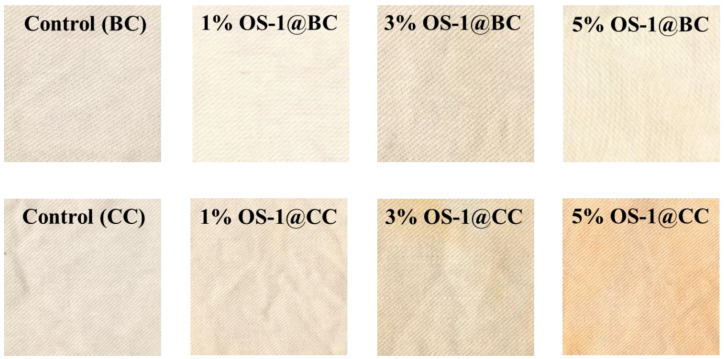

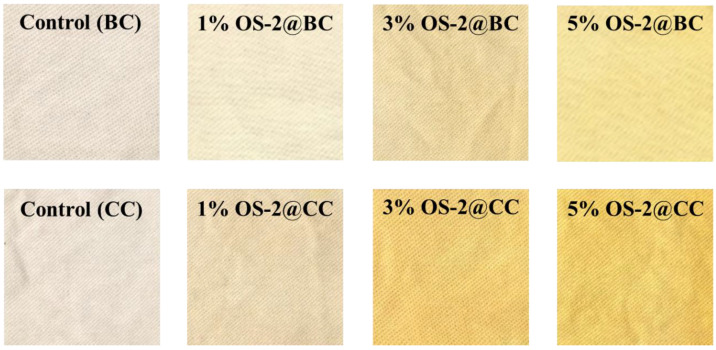
Digital photographs of control and OS-treated textiles.

Digital photographs of the untreated and OS-treated cotton textile samples are shown in Figure 6. The OS-1 treatment of the cotton textiles produced a reddish hue, whereas the OS-2 treatment of the cotton textiles produced a visible yellow color on the textile surfaces. The color development was concentration dependent and was more prominent when the same compound was applied to the CC than to the BC, for both OS-1 and OS-2. 

The evaluation of the color depth (color strength) of the cotton fabrics treated with the OS compounds was conducted through measuring the K/S value (where K is an absorption coefficient and S is a scattering coefficient) using a spectrophotometer. When the OS compounds were applied to fabric, the K/S increased, and, as expected, this increase was proportional to the concentration of the OS in the solution. 

**Figure 7:**
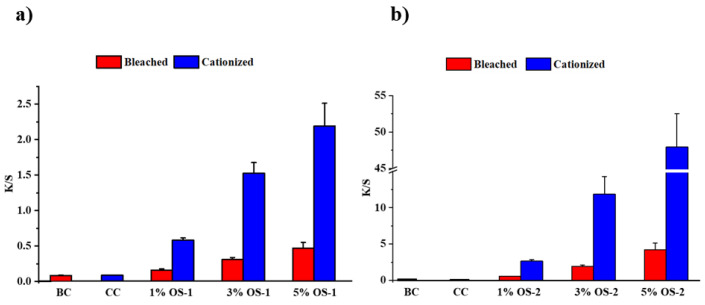
Color strength (K/S) of OS-1-treated textile (**a**), and OS-2-treated fabric (**b**). Values represent the means of triplicate experiments ± SD.

As can be seen from Figure 7, the K/S was determined to be 0.2 for the 1% OS-1-treated BC fabrics, 0.3 for the 3% OS-1-treated BC fabric, and 0.4 for the 5% treated BC fabric. There was a substantial increase in the K/S values when OS-2 was applied to BC when compared to those for OS-1. For the OS-2-treated fabrics, the K/S increased from 0.6 for the BC fabric treated with 1% OS-2 to 2.1 for the fabric treated with 3% OS-2 and 4.5 for the fabric treated with 5% OS-2. Overall, for the BC fabric treated with the OS compounds at the highest concentration (5%), a 10-fold increase in the K/S values between OS-1 and OS-2 was observed. 

A substantial increase in the K/S values was observed for the CC when compared to the BC, which is attributed to the additional ionic bonding of the CC with either of the OS compounds. Thus, the K/S for the OS-1-treated CC fabrics was found to be in the range from ~0.6 (for the fabric treated with the 1% solution of OS-1) to 2.2 (for the fabric treated with the 5% solution of OS-1). Substantially higher K/S values were obtained for the OS-2-treated fabrics: the K/S was found to be 2.5 for the fabric treated with 1% OS-2, 12.1 for the fabric treated with 3% OS-2, and 48.1 for the fabric treated with 5% OS-2. Overall, the K/S trend was as follows: OS-2@CC >> OS-1@CC > OS2@BC > OS-1@BC, independent of the concentration applied.

To describe the difference between the two colors, a color difference metric (**Δ**E*) was used in accordance with the International Commission on Illumination (CIE). The **Δ**E* levels are the difference between the color of the fabric and the reference color standard. Lower **Δ**E* values indicate similarity to the reference standard, whereas high **Δ**E* values indicate a significant deviation from the reference.

The **Δ**E* values of the OS-treated cotton were measured and compared with the reference textile; the results are given in Table 1. The OS-1 treatment of the BC textile produced a **Δ**E* between 3.40 and 12.38. Similar to the K/S study of the OS-treated textiles, the **Δ**E* values showed that the OS-2 treatment generated a more prominent color than the OS-1 treatment. Thus, the OS-2 treatment of the BC textile produced a **Δ**E* between 14.98 and 36.19, depending on the treatment concentration. A substantial increase in the **Δ**E* values was observed for the CC when compared to the BC. Thus, the OS-1 treatment of the CC textile yielded a **Δ**E* between 10.81 and 33.03, while the OS-2 treatment of the BC textile produced **Δ**E* values between 25.06 and 57.69, depending on the treatment concentration.

### 2.3. Textile Characterization

**Figure 8:**
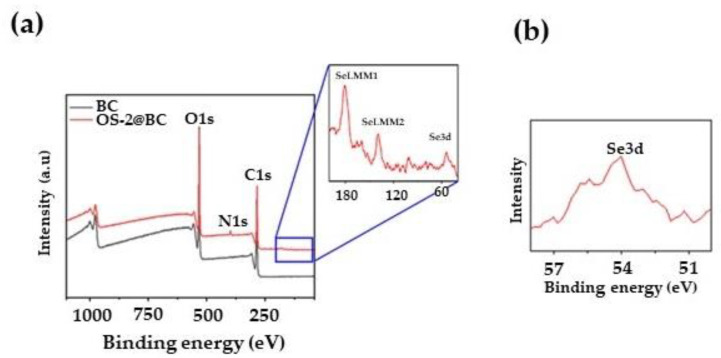
(**a**) Survey XPS spectra of control OS-2-treated cotton fabric and (**b**) high-resolution Se3d spectra of OS-2-treated cotton fabric.

FTIR was found to be not a sensitive enough technique for determining changes on the textile surfaces. Instead, for the surface characterization, X-ray photoelectron spectroscopy (XPS) was conducted on the fabric samples. All the samples were first analyzed through a survey scan (over a binding energy range from 0 to 1000 eV) to determine the elements present within the top 1–10 nm of the surfaces of the samples. Figure 8a shows the survey XPS spectra of the control (BC) and OS-2-treated cotton fabrics. Carbon and oxygen were found as expected for the cellulose, but there was also nitrogen observed in the OS-2-treated BC fabric. 

Namely, the survey XPS spectrum of the control fabric showed three peaks for O1s (532 eV), C1s (285 eV), and O2s (24.8 eV) orbitals [61]. Compared to the control fabric, the OS-2-treated fabric exhibited one additional peak N1s (398 eV) orbital [62]. Figure 8b shows the high-resolution Se3d spectrum of the OS-2-treated cotton fabric. A clear peak at 54 eV is obvious in the high-resolution spectrum, which can be attributed to the Se3d orbital [63]. These additional peaks in the OS-2-treated fabric confirm the presence of Se on the surface of the cotton fabric.

It is important to point out that, at this low concentration, these Selenium-based compounds behave more like reactive dyes than crosslinking agents. Typically, if crosslinking occurs between Se-based compounds and cellulose, FTIR spectra would show the presence of corresponding vibrations attributed to crosslinking agents. However, in our case, the FTIR did not show additional vibrations suggesting the occurrence of crosslinking. Further evidence for the absence of crosslinking is the higher reactivity of the Se-based compounds with the cationized cotton fabric as compared to that of the only bleached cotton fabric.

### 2.4. Antimicrobial Assays

After treatment, the fabric samples were tested for their antimicrobial activity using three strains of microorganisms: *S. aureus* AH133 GFP (Gram-positive), *Escherichia coli* MM294 (Gram-negative), and *Candida albicans* strain 3147 (fungi). S. *aureus* and *Escherichia coli* GFP lab strains were chosen as representatives of Gram-positive and Gram-negative bacteria. *Candida albicans* was chosen to represent a fungal species. 

In vitro CFU assays were conducted to determine the antimicrobial activity of the textiles. For conducting the assays, bacteria washed with phosphate-buffered saline (PBS) were suspended in PBS at a concentration of ~10^8^ CFU/mL, and then diluted with fresh PBS to a concentration of 10^2^–10^3^ CFU/mL (see Experimental for details). Fabric samples (1 cm^2^ area), 10 µL aliquots of inoculum, and 1 mL of fresh PBS buffer were placed into separate wells of a multi-well plate. After 24 h of incubation at 37 °C, the number of colonies formed on the fabrics was counted using dilution and plating techniques. The tests were conducted on the OS-1-treated BC and CC fabrics (OS-1@BC and OS-1@CC, respectively) and OS-2-treated BC and CC textiles (OS-2@BC and OS-2@CC, respectively). 

**Figure 9:**
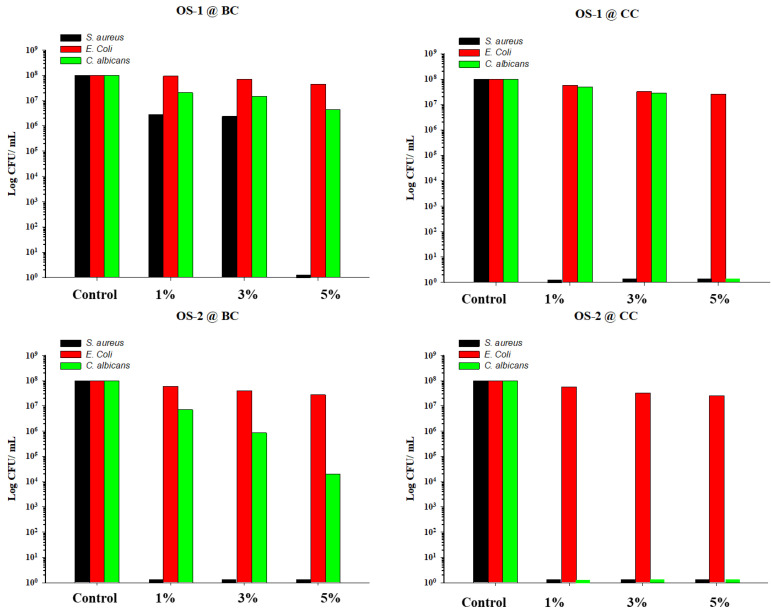
Graph of CFU/mL of *S. aureus*, *E. coli*, and *C. albicans* biofilms formed on the BC (control, **left**) and CC (**right**) textiles. Top: OS-1, Bottom: OS-2.

Initial screening was conducted on the fabrics treated with 1, 3, and 5% of OS-1 and OS-2, respectively, to determine the optimal concentration of the OS compounds. Two negative controls—untreated BC and CC fabrics—were also evaluated. Table 2 and Figure 9 summarize the antimicrobial activities of both the BC and CC OS-treated fabric samples, and the bacterial reduction is shown as both % reduction and log reduction.

The bleached fabrics treated with the 1% OS-1 solution allowed for a 97.24% reduction in *S. aureus* bacterial growth, while the fabrics treated with the 3% OS-1 solution were just slightly more active, yielding a 97.63% reduction in bacteria. With an increase in the OS-1 solution concentration to 5%, the inhibition effects on *S. aureus* raised to 100%. The bacterial count was significantly higher for the *E. coli* strain than that of *S. aureus*, indicating the lower effectiveness of OS-1 BC against *E. coli*. Thus, the BC fabric treated with 1% OS-1 yielded only a 6.06% *E. coli* bacterial reduction, the fabric treated with 3% OS-1 provided a 30.30% *E. coli* reduction, and the fabric treated with 5% OS-1 demonstrated 54.55% activity against *E. coli*. The activity against the *Candida albicans* strain was 79.35% at the lowest concentration of OS-1 (1%) and 95.60% at the highest concentration of OS-1 (5%). 

The BC fabric treated with OS-2 completely inhibited the growth of *S. aureus* at its lowest concentration (1% OS-2). Moderate activity, although higher than that in the case of OS-1, was observed against *E. coli*: 39.39%, 60.61%, and 72.73% for the 1, 3, and 5% OS-2-treated BC fabric, respectively. The antifungal activity was also higher in the case of the OS-2 treatment than that in the case of the OS-1 treatment, even though a complete inhibition of fungi growth was not achieved, and the bacterial reduction values ranged from 92.99% (1% OS-2 treatment) to 99.98 (5% OS-2 treatment).

The CC fabric treated with OS-1 completely inhibited the growth of the *S. aureus* bacteria at the lowest concentration (1% OS-1). The activity against *E. coli* was modest: 5% OS-1 killed 74.19% of the bacteria. A fungal reduction of 51.72% was found when the CC fabric was treated with 1% OS-1 and 72.41% when the CC fabric was treated with 3% OS-1, whereas 5% OS-1 yielded a complete elimination of bacterial growth. The CC fabric treated with OS-2 exhibited an excellent 100% bacterial reduction for both the *S. aureus* bacteria and *Candida albicans* yeast at its lowest concentration (1% OS-2). 

The observed improvement in the antimicrobial activity of OS-2 when compared to OS-1 was likely due to the abundance of Se atoms on the molecule of OS-2, whereas the enhancement in antimicrobial activity in the case of the CC vs. BC cotton was likely attributed to the higher fixation of OS molecules on the CC fabric. 

The differences in activities against the different species were likely due to the following. The reason for the high efficacy of OS against the *S. aureus* bacteria could be due to the abundance of Trx in their cells, which is an attractive target for Se-compounds [64,65]. It also appears that the OS compounds performed significantly better against *C. albicans*. Like bacteria, fungi also possess low MW Trx as a thiol-dependent ROS defense [66], and the antifungal mechanism of the OS compounds also involves the depletion of low MW thiols and the elevation of ROS. It is reasonable to speculate that there was a similar interaction between fungal Trx and the OS compounds to that in bacteria. An OS compound could also deplete fungal GSH [67].

All types of fabrics demonstrated moderate activity against the *E. coli* strains. The poor activity of OS against the *E. coli* strain could be attributed to its limited capacity to penetrate cell walls due to their outer membrane [68]. According to [69], the activity of OS compounds against *Escherichia coli* bacteria depends on the proportion of their accumulation to the polar amine group of the cell wall. This could be achieved by either introducing polar groups into the OS compounds or applying them in higher doses (>128 (μg/mL)) [69]. In addition, *E. coli* bacteria possess GSH as their major low MW thiol instead of Trx within their cell [65,70,71,72]. 

### 2.5. Leaching Studies

#### 2.5.1. Leaching (%) of OS from Treated Textile Due to Soaping

**Figure 10:**
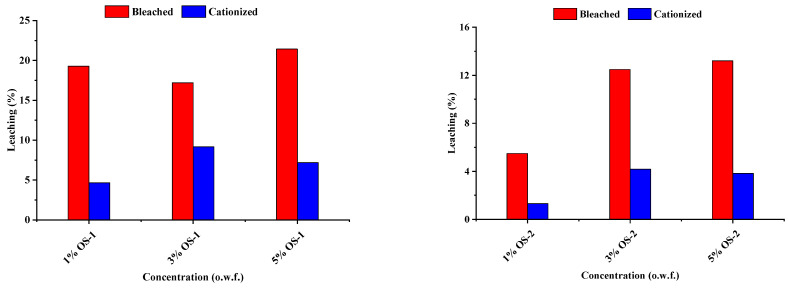
Leaching (%) of the OS compounds from treated textiles due to the soaping process.

The degree of leaching (in %) of the OS was determined after washing for 30 min in a non-ionic detergent (Triton X-100) at 100 °C and pH 8 (See Experimental). The degree of leaching (%) of the OS compounds during soaping from the treated textiles is shown in Figure 10. 

It is seen that the BC fabric treated with OS-1 released from 17 to 21% of active coating into a water phase, due to the washing with the non-ionic detergent. In contrast, the amount of OS-1 that leached out from the CC fabric was significantly less, from 5 to 9% OS-1. 

Among the two compounds, OS-2 was more resistant to leaching than OS-1. Thus, the BC fabric treated with OS-2 liberated less active leaching than the same fabric treated with OS-1, namely from 5 to 13%. Finally, the CC material treated with OS-2 was the most stable in terms of leaching, releasing from only 1 to 4% OS-2 into the aqueous phase. In general, a higher degree of leaching was observed for the bleached fabrics treated with OS compared to the cationized fabrics. This could be due to the better fixation of the OS compounds on the cationized fabric due to the positive amino groups on its surface. This improvement in the leaching resistance of the CC could be attributed to electrostatic interactions between the positively charged OS molecules and negatively charged cationized textile. This leaching behavior can be an indication of the wash fastness of OS-treated textiles. Further work can be conducted to improve the leaching stability or wash fastness behavior of OS compounds.

#### 2.5.2. Durability of Antimicrobial Activity

Textiles are frequently washed in the presence of soap or detergent over their life cycle. Antibacterial materials must be able to withstand repeated washing and laundering in domestic settings. To estimate the durability of the OS treatment of the cotton textiles, the 5% OS (o.w.f.)-treated textiles were washed using rigorous washing conditions (non-ionic detergent Triton X-100 and NaHCO_3_ at 100 °C for 30 min). The wash durability was measured in terms of in vitro CFU assays. 

**Figure 11:**
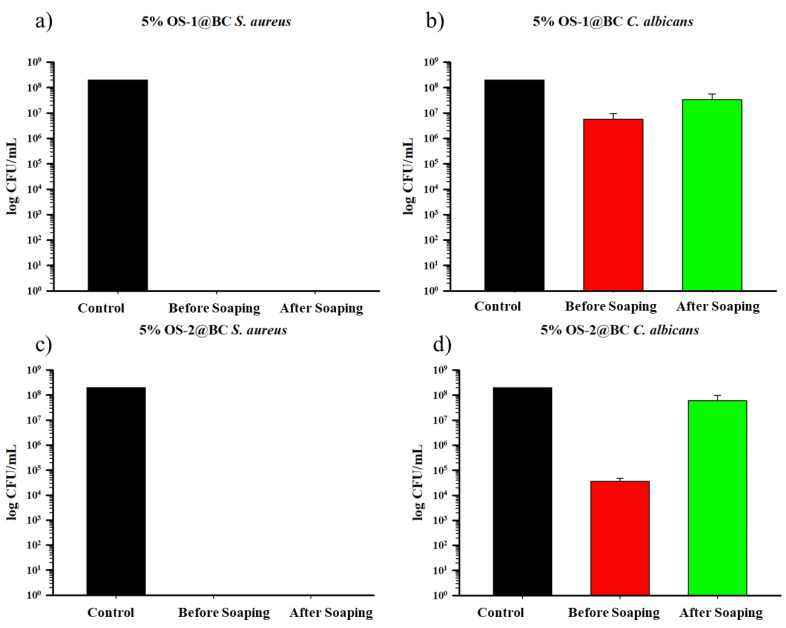
Graph of CFU/mL of *S. aureus* (**a**,**c**) and *C. albicans* (**b**,**d**) biofilms formed on BC textile treated with the OS compounds before and after the soaping process. Values represent the means of triplicate experiments ± SD.

**Figure 12:**
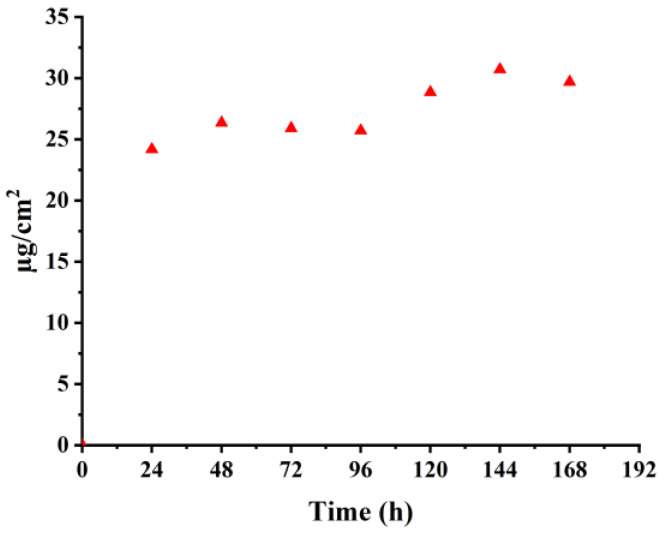
Release kinetics of textile sample 5% OS-2-treated BC.

The results of the in vitro CFU assays for the OS-treated BC textiles are shown in Figure 11, and for the OS-treated CC fabrics in Figure 12. For *S. aureus*, both the OS-1- and OS-2-treated BC fabrics, washed under rigorous conditions, retained their antimicrobial activity, as shown in Figure 11a,c, completely preventing bacterial growth either before or after washing. An apparent loss in the inhibition of *C. albicans* biofilms was seen for both the OS-1- and OS-2-treated BC fabrics washed under rigorous conditions (Figure 11b,d for OS-1 and OS-2, respectively), although the difference in the inhibition of *C. albicans* for the OS-2-treated BC fabric was larger than that for the OS-1 fabric. This indicates a loss of antifungal activity on the BC textile due to soaping. As was shown previously, the OS-treated BC fabric demonstrated little activity against the *E. coli* strains, which was not expected to change after washing, and the fabric was not re-tested against these bacteria.

The results of the in vitro CFU assays for the OS-treated CC textiles showed that, for *S. aureus*, both the OS-1- and OS-2-treated CC fabrics, washed under rigorous conditions, behaved similarly to the BC fabric and fully eliminated the *S. aureus* biofilm growth on both the unwashed and washed textiles. The same was true for *C. albicans* biofilms for both the OS-1- and OS-2-treated CC fabrics washed under rigorous conditions; a complete inhibition of *C. albicans* for both the OS-1- and OS-2-treated CC fabric was observed. This durability could be attributed to the strong bonding between the cationic amino groups and aromatic-ring-bearing OS molecules in pre-cationized cotton textiles. As reported earlier, the OS-treated CC fabric was only negligibly active against the *E. coli* strains, thus the rigorously washed fabric was not tested against this strain.

#### 2.5.3. Release of OS-2 from the Treated Fabric

To determine any potential release of Se from the treated textile into the environment, a leaching study was carried out. The aim of this part of the study was to quantify the release of OS-2 from the OS-2-treated BC (5% OS-2 concentration) into water. The reason behind choosing this particular material for the leaching studies was that OS-2 had a higher activity than OS-1, and its adherence to the BC fabric was weaker than that to the CC; hence, a larger amount of more active compound was expected to leach out.

A calibration curve was first obtained by plotting UV-Vis absorbances at the maximum absorbing wavelength (λ_max_) of a series of consecutively diluted OS-2 solutions in water at pH 7 as a function of concentration. An OS-treated BC fabric sample was placed into DI water and the OS-2 compound was allowed to leach. The UV-Vis absorbance was recorded every 24 h. The amount of released OS-2 was back-calculated from the calibration equation, considering the dilution factor. 

The release kinetics study showed that most of the compound (24 µg/cm^2^ of OS-2) was released within 24 h, as shown in Figure 12, after which, the release of OS-2 from the treated textile was slow. The highest amount of release of OS-2 (~31 µg/cm^2^) was obtained after 144 h of leaching. Because OS-2 is a newly synthesized compound and its minimum inhibitory concentration (MIC) and minimum biocidal concentration (MBC) are unknown, we cannot conclude whether the release of OS-2 was above the MIC and MBC of certain microorganisms in the water. 

## 3. Materials and Methods

### 3.1. Textile Material

Twill-weaved, desized, scoured, and bleached 100% cotton fabric was used for the research (warp 100 yarns per inch, weft 56 yarns per inch, 215 g/m^2^ pe meter). The fabric was supplied by Fiber and Biopolymer Research Institute, Lubbock, TX, USA. 

### 3.2. Chemicals

Acetic acid (CH_3_COOH, 99.7%) was obtained from Sigma Aldrich (St. Louis, MO, USA). Acetone (C_3_H_6_O, 99.5%), sodium bicarbonate (NaHCO_3_, 99.7%), sodium hydroxide (NaOH, 98%), and anhydrous sodium carbonate (Na_2_CO_3_, 99.5%) were obtained from Fisher Scientific (Hampton, NH, USA). A cationizing agent, 3-chloro-2-hydroxypropyltrimethyl ammonium chloride (CHPTAC), also called CR-2000 (C_6_H_15_Cl_2_NO, 65%), and Triton X-100 (2-[4-(2,4,4-trimethylpentan-2-yl)phenoxy]ethanol) (C_16_H_26_O_2_, 99+%) were obtained from Dow Chemical (Midland, MI, USA). Sodium sulfate anhydrous (Na_2_SO_4_, 99.5%) was received from Cooper Natural Resources (Fort Worth, TX, USA). The organoselenium compounds, OS-1 (C_6_Cl_4_N_6_Se_2_, >99%) and OS-2 (C_6_H_2_Cl_2_N_6_Se_4_, >99%), were obtained from Attach Chem (Lubbock, TX USA) and used as received. Deionized (DI) water was obtained from AquaOne (Amarillo, TX, USA) and was used for all purposes.

### 3.3. Bacterial Strains

*S. aureus* AH133 GFP (Gram-positive), *Escherichia coli* MM294 GFP (Gram-negative), and *Candida albicans* strain 3147 (ATCC 10231^TM^) were used in this study. Both the *S. aureus* AH133 GFP (Gram positive) and *Escherichia coli* MM294 GFP strains were available at the Texas Tech University Health Sciences Center, Lubbock, TX, USA. The *Candida albicans* strain 3147 (ATCC 10231^TM^) was purchased from ATCC Manassas, VA, USA. All the microorganisms were cultured in Luria-Bertani (LB) broth or LB agar plates at 37 °C. All the bacterial stock was preserved at −80 °C in an ultra-low-temperature (ULT) refrigerator.

## 4. Methods

### 4.1. Fabric Preparation

The cotton fabric was first washed in 2% (*v*/*v*) Triton X-100 (at a 1:100 liquor ratio) following the published procedure [73]. Specifically, a hot plate was used to heat and raise the temperature of the DI water (2.5 L) to 50 °C. At this point, 50 mL of Triton X-100 was added to the DI water and mixed well using a magnetic stirrer. Then, 25 g of fabric was immersed into the solution and stirred using a glass rod for 30 min. Then, the fabric was removed and rinsed five times with DI water. The rinsed fabric was dried in a laboratory oven at 80 °C for 60 min. The dried fabric was taken from the oven and then washed in a beaker containing pure acetone for 10 min (at 1:20 liquor ratio) at room temperature. Then, the fabric was removed and rinsed five times with DI water. After that, the rinsed fabric was again dried at 80 °C for 60 min in a laboratory oven. 

### 4.2. Cationization of Cotton Textile

The cotton fabrics were cationized using CR-2000 (65% aqueous solution of CHPTAC), a commercially available cationizing chemical, following a previously published study [59]. CR-2000 (17.5 g) was added to 1 L of DI water at room temperature. The cotton fabric (58.8 g) was immersed into the liquor under constant stirring. Then, NaOH (7.51 g) was added and the mixture was stirred constantly for 20 min. After 20 min, the fabric was taken out and squeezed by hand. The treated fabric was kept in a zipped plastic bag for 24 h to avoid evaporation of the cationizing agent. Then, the fabric was rinsed five times in DI water and neutralized in a 1 g/L acetic acid solution for 5 min at room temperature. Finally, it was again rinsed five times in DI water and dried at 80 °C for 60 min in a laboratory oven to obtain a cationic cotton fabric. 

### 4.3. OS Treatment of Textiles

#### 4.3.1. OS Treatment of Bleached Textile

The bleached cotton fabric was treated with 5% of the OS compound (on the weight of the fabric, o.w.f.) (at 1:40 material to liquor). Triton X-100 solution (200 mL of 0.2 g/L) was prepared at room temperature. The solution was heated to raise its temperature to 60 °C. The OS compound (250 mg) was added to the solution and constantly stirred for 20 min. The bleached cotton fabric (5 g) was immersed in the solution. After 20 min, 10 g of Na_2_SO_4_ was added to the bath under constant agitation. After 10 min, 1 g of Na_2_CO_3_ was mixed into the solution to adjust the pH to 11. The solution was maintained for a following 60 min under constant stirring. Then, the treated fabric was taken from the bath and rinsed five times in DI water to remove the unfixed OS compound from its surface. Then, acid neutralization was performed to remove the alkalinity. For the acid neutralization, the rinsed fabric was treated in a 0.5 g/L CH_3_COOH solution at room temperature for 15 min. Then, it was rinsed five times again in DI water. Finally, the material was squeezed by hand and dried in a laboratory oven for 30 min at 100 °C. 

#### 4.3.2. OS Treatment of Cationized Textile

To treat the cationized cotton fabric with 5% of an OS compound (o.w.f.), a similar route was followed to that above. The absence of Na_2_SO_4_ salt here was the only distinction. Briefly, 0.2 g/L of Triton X-100 solution (200 mL) was prepared in a beaker. The solution was then heated to raise the temperature to 60 °C. At 60 °C, the OS (250 mg) was added to the solution and stirred vigorously using a magnetic stirrer. After 20 min, a piece of cationized cotton fabric (5 g) was immersed in the bath and agitated for 20 min. At this stage, 1 g of Na_2_CO_3_ was added to the beaker to raise its pH to 11. The solution was held and stirred for 60 min. After that, the fabric was rinsed five times in DI water. Then, the rinsed fabric was acid neutralized in a 0.5 g/L acetic acid solution for 15 min at room temperature. The neutralized fabric was washed five times using DI water. After squeezing it by hand, the treated fabric sample was dried in an oven at 100 °C for 30 min. Four groups of samples were produced, and the samples were abbreviated as follows for a better understanding (Table 3).

### 4.4. CFU Antibacterial Assays

The stored bacteria (frozen stocks) were collected from the freezer and washed with phosphate-buffered saline (PBS) (pH 7.4). The washed bacteria were suspended in PBS to an optical density (OD_600_) of 0.42 to obtain approximately 10^8^ bacterial colonies (CFU) per milliliter. This original inoculum was serially diluted ten-fold (1:10 dilution) up to a dilution faction of 10^6^ (from approximately 10^2^ to 10^3^ CFU/mL) in PBS. The control (untreated) and OS-treated fabric samples were cut into small pieces with a 1 cm^2^ area each. Three pieces (3 × 1 cm^2^) of each sample were placed into separate wells of a multi-well plate. Then, 10µL aliquots containing from approximately 10^2^ to 10^3^ CFU/mL (original inoculum) and 1 mL of PBS were added to each well containing the fabric sample. The multi-well plate system was closed using the lid and incubated overnight in an oven at a 37 °C temperature. The next day, the fabric samples were collected from the wells and transferred to sterile microcentrifuge tubes containing 1 mL of PBS to collect the biofilm. The tubes were sonicated in a water bath sonicator for 5–10 min to loosen the biofilm on the surface of the fabric, and then strongly vortexed three times for 1 min to resuspend the bacterial cells. All the bacterial-cell suspensions were serially diluted 10-fold in PBS up to a dilution factor of 10^6^. Then, three drops of the 10 µL aliquots of each dilution were plated or spotted on LB agar plates. They were incubated overnight in an oven at 37 °C. All the assays were performed in triplicate. The following day, the total bacterial colonies were counted for three spots of a certain dilution and averaged to obtain a single *CFU*. The back-calculation for the bacterial colony was performed using Equation (1):

(1) CFUmL=CFU×Dilution factor×102

where A represents the number of colonies grown in the control sample and B represents the number of colonies grown in the treated sample.

### 4.5. Color Measurements

An X-Rite Ci7800 benchtop spectrophotometer (X-Rite, Inc., Grand Rapids, MI, USA) equipped with the Color iMatch program was used to measure the reflectance of the untreated control cotton fabrics (bleached and cationic) and OS-treated cotton fabrics. The instrument was initially calibrated using the manufacturer’s recommended two standard tiles (black and white). The color measurements were performed with a 25 mm aperture using one fabric layer. Four measurements were made from each sample (different locations along the front and back side of the control and treated samples) under D65 illumination and 10° standard observer for the wavelength range from 360 to 750 nm. The Color iMatch program provided the average reflectance (%) values for each sample between 360 and 750 nm. The average relative color strength (*K*/*S*) values of the control and treated fabric samples were also obtained from the Color iMatch program. This program uses the Kubelka–Munk formula to give relative color strength (*K*/*S*) values, as shown in Equation (2):

(2) $$\frac{K}{S}=\frac{{\left(1-R_{\lambda }\right)}^{2}}{2R_{\lambda }}$$

where $R_{\lambda }$ is the reflectance value at the minimum reflecting wavelength (λ_min_), *K* is the absorbing coefficient, and *S* is the scattering coefficient.

The CIE L*, a*, and b* values of the control and OS-treated cotton fabrics were also collected from the Color iMatch program. This program provided the averaged CIE L*, a*, and b* values of the four readings from each sample. Here, L* is defined as lightness. The value of L* can vary between 0 and 100, where 0 means black and 100 means white. The value of a* expresses the redness or greenness of a sample. The positive and negative values of a* indicate the redness and greenness, respectively. The value of b* expresses the degree of the yellowness or blueness of a sample. The positive and negative values of b* indicate the yellowness and blueness, respectively. The color difference (ΔE*) between the reference (untreated) fabric and OS-treated fabric was measured using Equation (3):

(3) $$\Delta E^{*}=\sqrt{{\left(\Delta L^{*}\right)}^{2}+{\left(\Delta a^{*}\right)}^{2}{+(\Delta b^{*})}^{2}}$$

where ΔL*, Δa*, and Δb* are the differences between the color coordinates of the reference and tested fabric samples.

### 4.6. Fixation of OS to the Textile

The fixation (%) of the OS compound on the cotton textile was measured from the relative difference in the concentration of the OS solution before and after the treatment. The absorbances of the treatment solution were measured, before and after the treatment, using a Perkin Elmer Lambda 650 UV/Vis Spectrophotometer (PerkinElmer, Inc., Waltham, MA, USA). Using the calibration curve and Beer–Lambert law, the absorbances were converted into concentrations, and the fixation (in %) was calculated using Equation (4):

(4) $$\%\;F=\frac{C_{o}-C_{t}}{C_{t}}\times 100$$

where *C_o_* is the concentration of the OS solution before treatment and *C_t_* is the concentration of the OS solution after treatment.

### 4.7. Release of OS from Treated Textile

A release study of the OS-treated fabric samples in DI water (1 to 50 liquor ratio) per unit area of fabric was conducted using the Ohaus Orbital Shaker (Ohaus Corp., Parsippany, NJ, USA). A 5% OS-2 (o.w.f.)-treated cotton fabric with a 6 cm x 6 cm area was placed in a conical flask containing 50 mL of DI water at room temperature. The flask was then placed in the Ohaus Orbital Shaker (Ohaus Corp., Parsippany, NJ, USA) and continuously shaken at 150 RPM. At every 24 h interval, 3–4 mL of solution was collected from the flask and transferred to a cuvette to collect the absorbance. The absorbance readings at the maximum absorbing wavelength (λ_max_) were taken using a Perkin Elmer Lambda 650 UV/Vis Spectrophotometer (PerkinElmer, Inc., Waltham, MA, USA). The solution was again placed back into the flask to maintain the initial liquor ratio (1:50). The experiment was run for 168 h. Finally, the release of the OS compound was calculated in µg/cm^2^ of the treated fabric and plotted in a graph as a function of time.

### 4.8. Leaching of OS Due to Soaping

**Figure 13:**
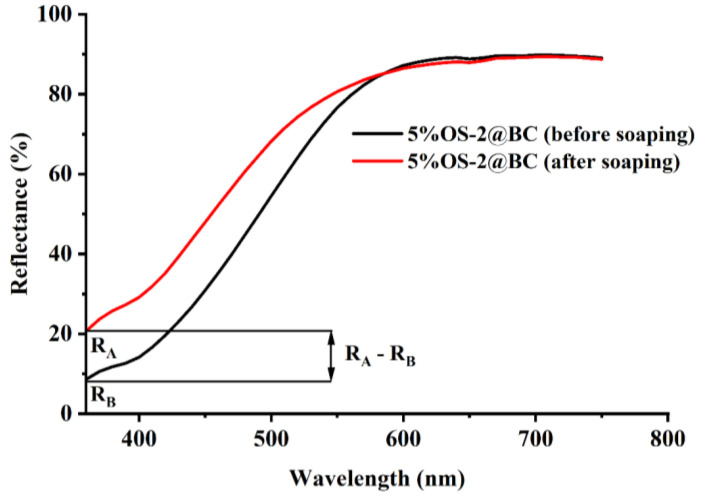
Difference in reflectance of an OS-2-treated textile before and after soaping procedure.

The leaching of the OS due to washing with a soaping agent under strong washing conditions was performed. For the leaching study, OS-treated fabric samples were first soaped following the procedure reported in this reference [74]. Briefly, the OS-treated fabric samples were washed (at a 1: 100 liquor ratio) using 2 g/LL of Triton X-100 and 5 g/LL of NaHCO_3_ at 100 °C for 30 min. After washing, the fabric was rinsed five times in DI water and dried in a laboratory oven at 80 °C for 30 min. The reflectance of the textiles (before and after the soaping process) was taken using an X-Rite Ci7800 benchtop spectrophotometer (X-Rite, Inc., Grand Rapids, MI, USA). The degree of leaching (% *L*) was calculated from the minimum of the reflectance spectrum (λ_min_) using Equation (5) [75]:

(5) $$\%\;L=\frac{R_{A}-R_{B}}{100-R_{B}}\times 100\;\%$$

where $R_{B}$ represents the reflectance of the treated fabric before soaping and $R_{A}$ represents the reflectance of the treated fabric after soaping. *L* = 100% denotes a totally decolorized cloth, whereas *L* = 0 denotes the material as it was originally colored (Figure 13)

### 4.9. Durability of OS-Treatment

To study the durability of the treatment, 5% OS-treated fabric samples were soaped using 2 g/L of a non-ionic detergent (Triton X-100) and 5 g/L of NaHCO_3_ at 100 °C for 30 min. They were rinsed several times followed by drying in a laboratory oven at 80 °C for 30 min. The soaped samples were again tested for their antibiofilm activity. The numbers of biofilms formed on the textiles, before and after the soaping, were compared to determine the durability of the OS treatment.

### 4.10. Fourier Transform Infrared Spectroscopy (FTIR) 

The FTIR spectra of the fabric samples were collected using FTIR spectroscopy (Spectrum 400, PerkinElmer, MA, USA) equipped with a ZnSe diamond crystal and pressure arm. Three spectra were collected from each sample at a spectral resolution of 4 cm^−1^ and 64 co-added scans in the range of 4000–650 cm^−1^. 

### 4.11. X-ray Photoelectron Spectroscopy (XPS)

Initially, the bleached cotton was treated with 2% OS-2 and sent to the Materials Characterization Center, Edward E. Whitacre Jr. College of Engineering, TTU, for an XPS analysis. The XPS study of the control and OS-2-treated fabric was performed to determine the elemental composition of the fabric surface using a PHI 5000 VersaProbe-II Hybrid X-ray photoelectron spectrometer (Physical Electronics, Inc., Chanhassen, MN, USA). This instrument has an operating pressure of 5 × 10^−7^ Pa and minimum probe size of 10 µm. The source electron beam is generated by an LaB6 filament and focused using an electrostatic lens. On the aluminum (Al) anode, a scanning Al Kα X-ray is generated by the scanning electron beam. A quartz crystal monochromator reflects the monochromatic Al Kα scanning X-ray onto the sample as a source beam. When photo electrons are generated by the X-ray and pop out of the surface, an analyzer lens makes an emission angle of 45° to enhance the number of photoelectrons reaching into the analyzer. A hemispherical spherical analyzer filters out the photo electrons of the set energies to finally reach the detector. A dual-beam charge neutralizer was used to eliminate the samples’ static charge.

## 5. Conclusions

Reactive chlorotriazinyl fragments were incorporated into the structure of two novel organoselenium compounds, namely 1,2-bis(4,6-dichloro-1,3,5-triazin-2-yl)diselane (OS-1) and 6,6′-diselanediylbis(4-chloro-1,3,5-triazine-2-selenol) (OS-2), which allowed for their covalent attachment to cotton cellulose via the nucleophilic aromatic substitution (S_N_Ar) reaction. According to in vitro CFU assays, the OS compounds were strongly active against the Gram-positive *S. aureus* strain, fully eliminating bacterial growth on these fabrics after the treatment. The inhibition of *C. albicans* biofilms proceeded to a lower extent on the bleached cotton, but fully eliminated the bacterial growth on the cationized substrate. The OS-treated fabric demonstrated modest activity against the *E. coli* strains. In all cases with detected antimicrobial activity, the OS-treated cationized fabric exhibited higher activity than the bleached textile, and OS-2 was significantly more active than OS-1.

After washing under rigorous conditions to assess the durability of the treatment, both the OS-1- and OS-2-treated BC fabrics retained the antimicrobial activity against *S. aureus*, completely preventing bacterial growth. A slight loss in antifungal activity against *C. albicans* was observed, more so for the OS-2-treated BC fabric than for the OS-1-treated cotton. For the CC fabrics, both the OS-1- and OS-2-treated textiles fully eliminated the *S. aureus* and *C. albicans* biofilm growth on the washed textile. The durability of the treatment in the case of the CC could be attributed to the strong bonding between the cationic amino groups and aromatic-ring-bearing OS molecules. In summary, these reactive organo-selenium compounds evaluated as an efficient route for producing antimicrobial textiles did not interrupt the regular production processes of a textile mill.

## 6. Patents

The work presented here is under consideration by the World Intellectual Property Organization WO 2023/059540 A1.

## Figures and Tables

**Table 1:** CIE L*, a*, b*, and ΔE* values of the control and OS-treated textiles.

Bleached Cotton				
Sample Name	a*	b*	L*	ΔE*
Control (BC)	−0.31	2.61	95.50	0
Control (CC)	−0.26	2.56	95.58	0
Bleached Cotton/OS-1				
1% OS-1@BC	−0.05	5.30	94.81	3.40
3% OS-1@BC	1.85	13.64	92.12	14.12
5% OS-1@BC	0.70	12.42	93.02	12.38
Cationized Cotton/OS-1				
1% OS-1@CC	2.29	14.16	91.66	14.98
3% OS-1@CC	4.12	22.86	89.23	26.16
5% OS-1@CC	12.31	28.23	82.69	36.19
Bleached Cotton/OS-2				
1% OS-2@BC	−1.69	10.67	94.40	10.81
3% OS-2@BC	0.08	27.77	90.57	32.55
5% OS-2@BC	1.79	28.04	89.26	33.03
Cationized Cotton/OS-2				
1% OS-2@CC	−1.09	22.57	89.17	25.06
3% OS-2@CC	5.14	44.73	85.07	53.24
5% OS-2@CC	6.25	48.14	82.97	57.69

L* represents a lightness difference, a* represents the difference in redness or greyness, and b* denotes blueness-yellowness differences between the sample and standard colors.

**Table 2:** Antimicrobial activity of control (bleached) and OS-1-treated fabrics.

Sample Name	*S. aureus*	*Escherichia coli*	*Candida albicans*	*S. aureus*	*Escherichia coli*	*Candida albicans*
	Percent Reduction (%)			Log Reduction ^a^		
Control (BC)	0	0	0	0.00	0.00	0.00
Control (CC)	0	0	0	0.00	0.00	0.00
OS-1						
Bleached Cotton, BC						
1% OS-1	97.24	6.06	79.35	1.56	0.03	0.69
3% OS-1	97.63	30.30	85.00	1.63	0.16	0.82
5% OS-1	100.00	54.55	95.60	8.00	0.34	1.36
Cationized Cotton, CC						
1% OS-1	100.00	43.55	51.72	8.00	0.25	0.32
3% OS-1	100.00	67.74	72.41	8.00	0.49	0.56
5% OS-1	100.00	74.19	100.00	8.00	0.59	8.00
OS-2						
Bleached Cotton, BC						
1% OS-2	100.00	39.39	92.99	8.00	0.22	1.15
3% OS-2	100.00	60.61	99.15	8.00	0.40	2.07
5% OS-2	100.00	72.73	99.98	8.00	0.56	3.70
Cationized Cotton, CC						
1% OS-2	100.00	43.55	100.00	8.00	0.25	8.00
3% OS-2	100.00	67.74	100.00	8.00	0.49	8.00
5% OS-2	100.00	74.19	100.00	8.00	0.59	8.00

^a^ Log Reduction = −(log_10_(−1 × Percent Reduction/100 + 1)). A 1-log reduction corresponds to inactivating 90 percent of a target microbe, with the microbe count being reduced by a factor of 10. Thus, a 2-log reduction corresponds to a 99 percent reduction, or microbe reduction by a factor of 100, and so on.

**Table 3:** Sample name and details.

Abbreviation	Description
BC	Bleached cotton fabric
CC	Cationized cotton fabric
5%OS-1@BC	5% o.w.f. OS-1 applied to a bleached cotton fabric
5%OS-2@BC	5% o.w.f. OS-2 applied to a bleached cotton fabric
5%OS-1@CC	5% o.w.f. OS-1 applied to a cationized cotton fabric
5%OS-2@CC	5% o.w.f. OS-2 applied to a cationized cotton fabric

o.w.f. = on the weight of the fabric.

## Data Availability

Not applicable.
